# Supplementary material for: Impact of stain variation and color normalization for prognostic predictions in pathology
Source: Sci Rep. 2025 Jan 18;15:2369. doi: 10.1038/s41598-024-83267-w (PMC11742970; doi:10.1038/s41598-024-83267-w)
Supplement: Supplementary file 1 — Supplementary Tables. [file 41598_2024_83267_MOESM1_ESM.docx]

**Supplemental Information: Impact of Stain Variation and Color Normalization for Prognostic Predictions in Pathology**

**Supplemental Table S1**

**
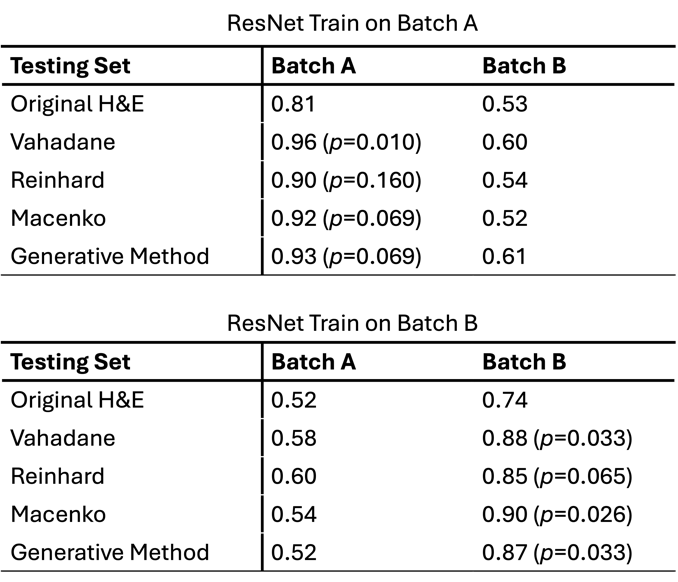
**

*Supplemental Table S1. Results summary of all stain-normalization methods where we train the ResNet models on either batch and test the models within the same-batch or cross-batch. We perform hypothesis testing where the null hypothesis states that the model testing accuracy with color normalization is the same as using just the original H&E images and the alternative hypothesis as the model testing performance with color normalization is better than with the original H&E images. p-values are indicated in the parenthesis.*

**Supplemental Table S2**

**
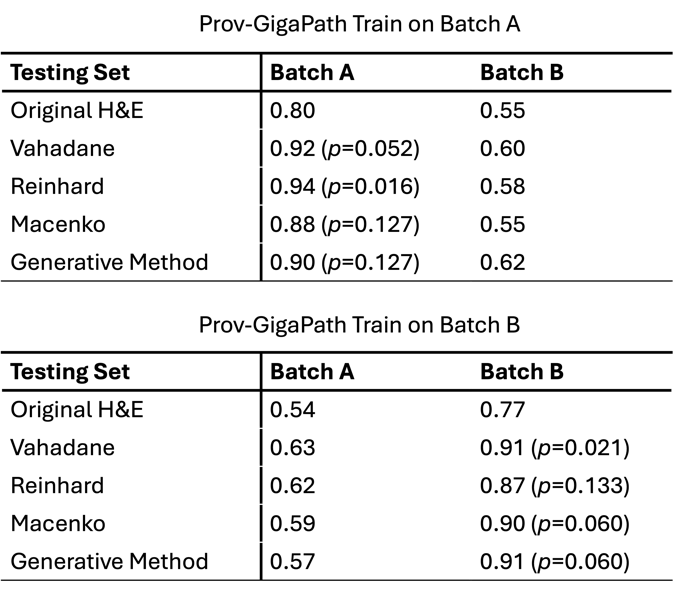
**

*Supplemental Table S2. Results summary of all stain-normalization methods where we train the Prov-GigaPath models on either batch and test the models within the same-batch or cross-batch. We perform hypothesis testing where the null hypothesis states that the model testing accuracy with color normalization is the same as using just the original H&E images and the alternative hypothesis as the model testing performance with color normalization is better than with the original H&E images. p-values are indicated in the parenthesis.*
